# Forearm Motion Recognition With Noncontact Capacitive Sensing

**DOI:** 10.3389/fnbot.2018.00047

**Published:** 2018-07-27

**Authors:** Enhao Zheng, Jingeng Mai, Yuxiang Liu, Qining Wang

**Affiliations:** ^1^The State Key Laboratory of Management and Control for Complex Systems, Institute of Automation, Chinese Academy of Sciences, Beijing, China; ^2^The Robotics Research Group, College of Engineering, Peking University, Beijing, China; ^3^The Beijing Innovation Center for Engineering Science and Advanced Technology (BIC-ESAT), Peking University, Beijing, China

**Keywords:** noncontact capacitive sensing, upper-limb motion recognition, human-machine interface, automatic data labeling, robot learning from humans

## Abstract

This study presents a noncontact capacitive sensing method for forearm motion recognition. A method is proposed to record upper limb motion information from muscle contractions without contact with human skin, compensating for the limitations of existing sEMG-based methods. The sensing front-ends are designed based on human forearm shapes, and the forearm limb shape changes caused by muscle contractions will be represented by capacitance signals. After implementation of the capacitive sensing system, experiments on healthy subjects are conducted to evaluate the effectiveness. Nine motion patterns combined with 16 motion transitions are investigated on seven participants. We also designed an automatic data labeling method based on inertial signals from the measured hand, which greatly accelerated the training procedure. With the capacitive sensing system and the designed recognition algorithm, the method produced an average recognition of over 92%. Correct decisions could be made with approximately a 347-ms delay from the relaxed state to the time point of motion initiation. The confounding factors that affect the performances are also analyzed, including the sliding window length, the motion types and the external disturbances. We found the average accuracy increased to 98.7% when five motion patterns were recognized. The results of the study proved the feasibility and revealed the problems of the noncontact capacitive sensing approach on upper-limb motion sensing and recognition. Future efforts in this direction could be worthwhile for achieving more promising outcomes.

## 1. Introduction

Emerging robotic technologies that can augment, replace or imitate the functions of human upper limbs are attracting greater attention in the field of industrial manufacturing. For instance, robot learning from humans (Billard et al., 2016), which aims to automatically transfer human motor skills to robots, rather than by manual programming, could greatly increase the working efficiency of industrial robotic control. To accomplish this goal, an important step is to accurately recognize the motions of the operator to instruct the robotic controller. The sensing method is the first key factor that determines the following process of human motion recognition (Pons, 2008; Tang, 2016). Optical-based methods (Bruno and Khatib, 2016) (cameras, lasers, and depth sensing technologies, etc.) and mechanical sensing methods (Dipietro et al., 2008; Xsens, 2017^1^) (motion capture system, inertial sensing technologies, and data gloves, etc.) are widely used both in academic research and commercial products, as the signals convey abundant human motion information. However, these sensing methods cannot obtain human intent information from muscle contractions.

Muscles are the actuators of human motion and act according to neural stimulations (Winter, 2009). Muscle contractions lead to joint intuitive motions, rather than responding to them. Compared with motion measurement signals such as the inertial measurement unit (IMU), muscle contraction signals not only contain the kinematic information, such as posture and motion patterns, but also the dynamic information such as the joint force and joint stiffness (Ajoudani, 2016). They offer opportunities to obtain more abundant human motion information and a faster time response. Many researchers have extracted human motion information from muscle signals to build a human-machine interface for robotic control. In the current technology, the surface electromyography (sEMG) signals are the most widely used for human motion recognition. sEMG can be measured in a non-invasive way with a relatively stable quality in rigorous human motions. When converting the sEMG signals to the corresponding human motion information, the two main methods being studied are the machine learning based method and the model based method. In the former method, the performance mostly relies on the statistical representations of the sEMG features. With a proper training procedure, it has been shown to produce relative stable recognition performances (accuracies) on multiple individuals (Novak and Riener, 2015). It has also been studied for robotic manipulator control (Artemiadis and Kyriakopoulos, 2010; Liarokapis et al., 2013; Murillo and Moreno, 2016). For instance, in a study published by Artemiadis and Kyriakopoulos (Artemiadis and Kyriakopoulos, 2010), a robotic manipulator control method based on sEMG signals was proposed. The design of the method was based on a support vector machine (SVM) to continuously map the joint positions with sEMG features, and the position errors could be controlled within several centimeters. The second type of method is the model based method, which takes advantage of the physical significance of the sEMG signals. It is also widely studied in robotic manipulator control (Ikemoto et al., 2015; Ison et al., 2016; Lunardini et al., 2016). For example, Lunardini et al. (2016) proposed a 2-degree-of-freedom (DOF) robotic joint torque control method based on the muscle synergy model. In the model, a synergy matrix was designed to map the N-dimensional neural activations with the M-dimensional sEMG signals. The robotic joint torques could be calculated from the difference between the flexion and extension of neural activations. The effectiveness of this method was validated by using an inter-day experiment and the robustness was proved to be better than the traditional muscle-pair methods.

The sEMG-based studies provided a promising path toward real applications of a human-machine interface. However, as highlighted in many related studies, limitations still exist. Firstly, the sEMG signals are measured using the electrodes that are placed on specific sites. In order to obtain a better signal quality, the electrodes have to firmly adhere to the skin. Sweat on the surface of the skin seriously decreases the signal quality and the subsequent recognition performance (Sensinger et al., 2009). Secondly, the sensing positions of the sEMG electrodes influence the recognition performance and therefore, a configuration procedure is needed before each use (Young et al., 2011). To overcome these drawbacks, some researchers have proposed adaptive recognition methods (Gijsberts et al., 2014; Zhai et al., 2017) or the use of high density electrodes (Ison et al., 2016). However, it is still challenging to meet the high demand of human motion recognition in practical applications.

Attempts are being made by using other signal sources to bypass the limitations in sEMG. The muscle contractions not only generate electric effects (recorded as sEMG signals), but also produce shape changes. Studies using ultrasound muscle imaging have proved that the muscle morphological parameters, such as the muscle thickness and pennation angle correlate with joint motion information (Shi et al., 2008; Zhou, 2015). Some researchers have used ultrasound muscle imaging signals for human motion recognition. For instance, Castellini et al. (2012) predicted finger position changes based on features calculated from latitudinal ultrasound images of the forearm. A strong linear relationship was found between the features and motions. On the other hand, the drawbacks of the technique are obvious. During measurements, conductive cream is needed at the sensing sites to insure the signal quality. Displacement between the probe and human skin also negatively affects the performance. Although ultrasound muscle signals are not suitable for human motion recognition in their current state. The studies revealed that muscle shape change information could potentially be used as a signal source for human motion recognition.

In our previous works (Zheng et al., 2014; Zheng and Wang, 2017; Zheng et al., 2017a), we proposed a noncontact capacitive sensing method for human motion recognition. The method measured the limb shape changes during human motion using a set of capacitors, and the sensing front-ends of the system were not in contact with human skin. It was proved in previous studies that noncontact capacitive sensing could produce comparable recognition results with sEMG-based strategies for lower-limb motion recognition. The capacitive sensing approach is a promising alternative solution to sEMG-based methods for human motion recognition. However, it has not been systematically studied for upper-limb motions. Upper limb motion patterns are different to lower limb motion patterns, with tasks being more dexterous with no periodicity. The muscle volumes are also smaller than that of the lower limbs. As the sensing principle of capacitive sensing is to measure limb shape changes caused by muscle deformations, the methods for lower-limbs, such as gait-phase-based (Zheng and Wang, 2017; Zheng et al., 2017a) cannot be used for upper limb motion recognition. Therefore, upper-limb motion recognition with capacitive sensing needs to be explored.

The contributions of the study are two fold. Firstly, we provide an alternative solution to the existing studies in upper-limb motion recognition tasks. We aim to recognize upper-limb motion patterns from muscle contraction signals using a noncontact method. Secondly, we aim to broaden the application of the capacitive sensing method to new tasks. The issues regarding the use of the methods for upper limb motion sensing and recognition are addressed. We recently made some initial attempts on upper-limb motion recognition using the capacitive sensing method (Zheng et al., 2017b). However, in the work of (Zheng et al., 2017b), the sensing approach was only implemented on one subject with static motion recognition (without motion transitions). Further critical problems that are relevant for practical use have yet to be addressed. In this study, we proposed a noncontact capacitive sensing method for upper-limb motion recognition. The sensing front-ends and the recognition method were designed and the confounding factors in data segmentation, training procedure and motion types were evaluated. Moreover, the method for automatic data labeling was designed to accelerate the training procedure.

## 2. Methods

### 2.1. Measurement system

**Figure 1:**
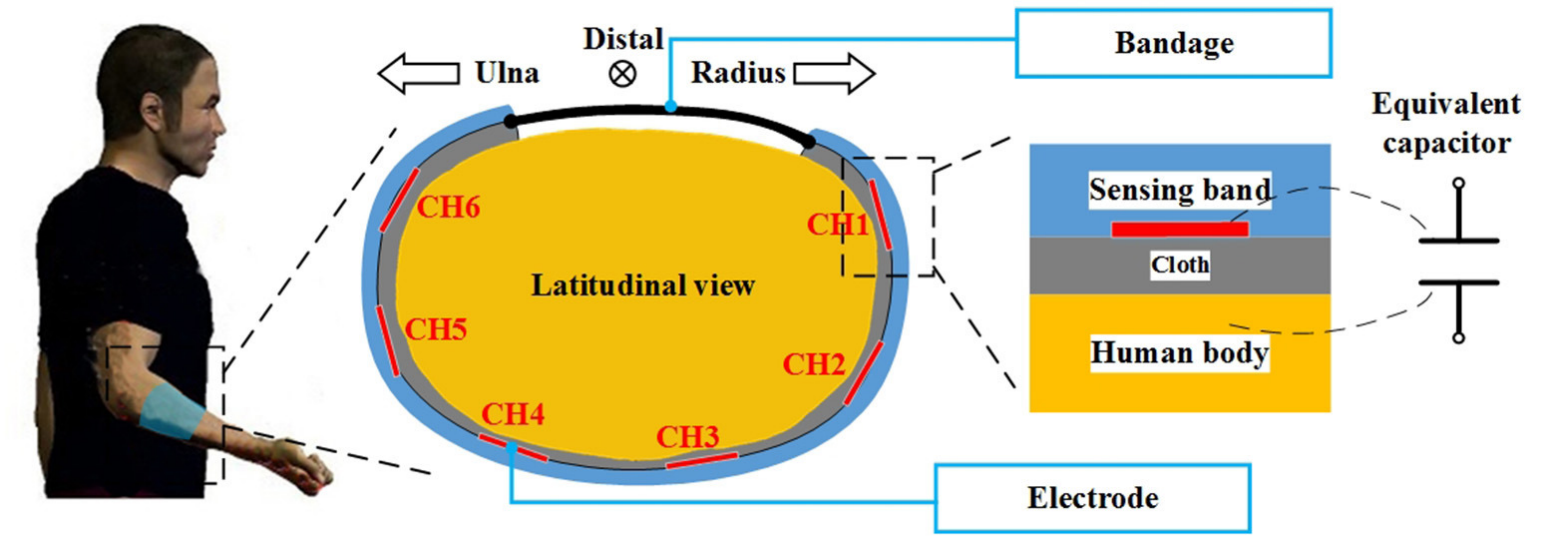
The sensing principle of the capacitive sensing system. The sensing front-end was placed onto the forearm of the human body. As shown in the figure, the sensing front-end was a C-shaped band which encircled the arm. The bandage was applied to the gap in the band to adjust the tightness. The electrodes were fixed on the inner surface of the band, an expansion of this can be seen on the right side of the figure. There were layers of cloth between the electrode and the human body. Using this configuration, the electrode, cloth and human body formed an equivalent capacitor.

The measurement system comprised of a capacitive sensing front-end, a capacitive sensing circuit (or capacitive signal sampling circuit), an IMU and a control circuit. The capacitive sensing front-end was designed to be applied onto the forearm to measure the muscle shape changes during motion. The main structure of the front-end was a band made of a thermoplastic material. The material became soft when heated up to 70°C and recovered at normal temperature. This characteristic made it possible to customize the size of the sensing front-end for each subject. The sensing-band was an open circle and the gap was placed on the medial side of the limb. A bandage was applied onto the edge of the gap for adjusting the tightness of the sensing band when fitting. Six copper films were fixed onto the inner surface of the sensing band which served as the electrode of the capacitive sensing system. The front-end was worn outside of the clothes (see Figure 1). When being fitted onto the human body, each copper electrode, the human body, and the cloth between them formed a plate capacitor. When limb shape changes occurred, the distance between the human skin and the electrodes changed, which caused a change to the capacitance value. By recording the capacitance signals, we could obtain upper-limb motion information. The capacitive sensing circuit was designed to measure capacitance values. A time-to-digital model was integrated into the circuit to record the charge-and-recharge cycle time of the equivalent capacitors. In order to convert the time to actual capacitance values, a reference capacitor was embedded onto the circuit. Using this method, the cycle time ratio between the reference capacitor and each equivalent capacitor (capacitive sensor) could be used to derive the actual capacitance values. In the initial attempts, the reference capacitor was set as 100 pF. The capacitive sensing circuit could sample capacitance values from 12 to 800 pF, more details can be found in Zheng et al. (2014). In this study, an IMU module was placed on the back of the measured hand to record the Eular angles (pitch, roll, and yaw). The output range of each axis was from –180 to 180°. For both of the circuits (the capacitive sensing circuit and the IMU module), the sampling rate was 100 Hz. The control circuit was designed to synchronize the capacitance signals and the IMU signals. All of the sensor data were packaged and transmitted to the computer every 10 ms (the sampling rate was also 100 Hz). We also designed a graphic user interface (GUI) on the computer to control data communication and conduct the experiment.

### 2.2. Experiment setups

#### 2.2.1. Motion recognition

**Figure 2:**
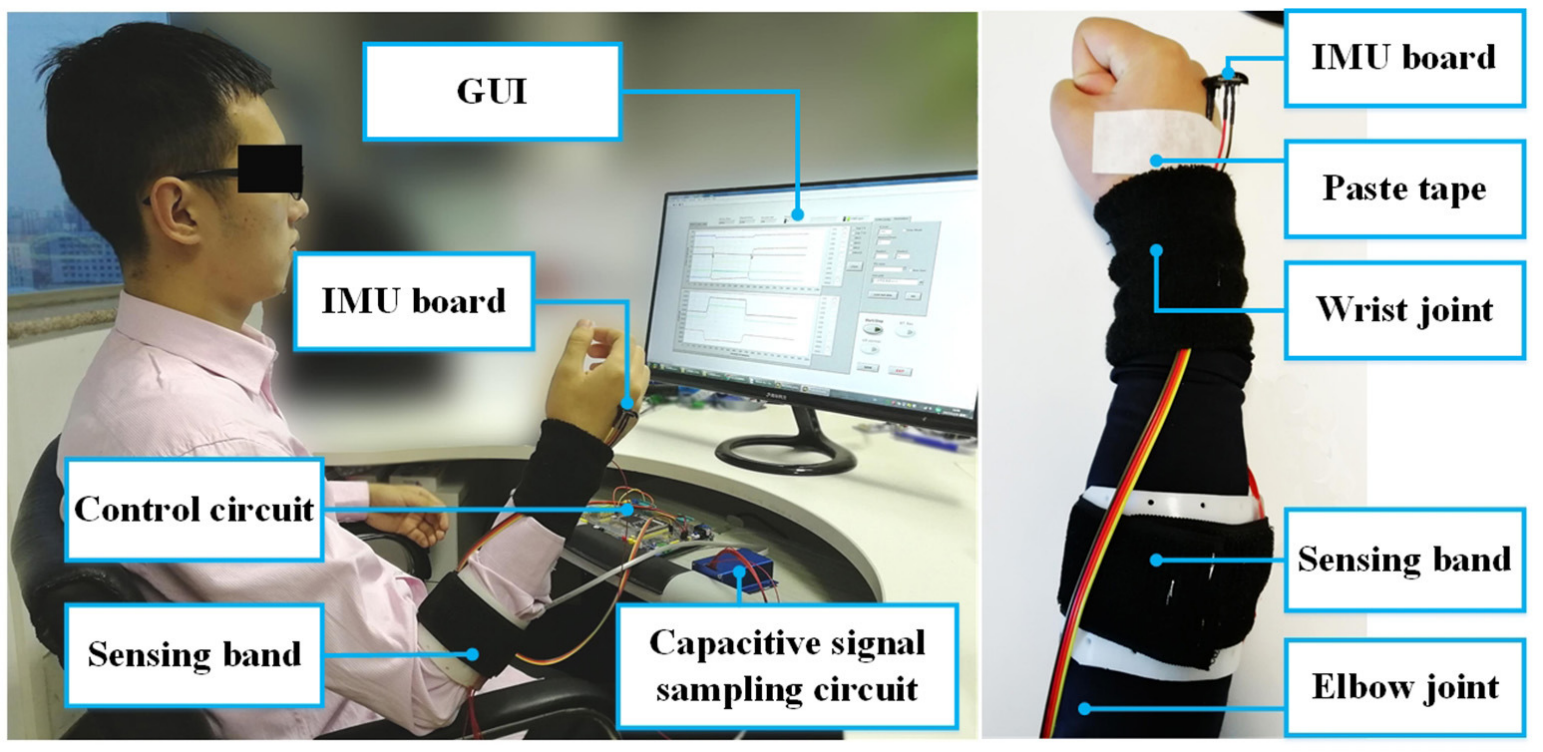
Experimental setup **(left)** and placement of the capacitive sensing band on human body **(right)**. The communication between the circuits and the computer was accomplished through the serial port. The capacitive signal sampling circuit was fixed inside a specifically designed metal box for electromagnetic shielding, shown as the blue box in the figure. During the experiment, the subject sat in front of the screen and performed the motion tasks following instructions. The experimenter conducted the experiment (not shown in the figure). The IMU board was pasted on the back of the measured hand to record the Eular angles. The figure shows subject 2 (S2), the subject gave written informed consent for the publication of this image.

The first experiment (Exp 1) conducted was the motion recognition experiment. Seven healthy participants were recruited for the experiment. All of the participants provided written and informed consent. The experiment was approved by the Local Ethics Committee of Peking University. The demographic information of the subjects is shown in Table 1. In this table, FL represents the forearm length (measured arm), which is the length between the stylion radiale and the radiale (elbow joint) with the arm sagging naturally. The abbreviation FC was used for forearm circumference which was measured from the most prominent part of the forearm. Before each subject took part in the experiment, a sensing front-end was customized based on the shape of the forearm. The sensing band was worn on the most prominent part of the measured forearm, which was about two thirds of the total forearm length from the distal end. The length of the sensing band was built based on the FL of each subject, the distance of the gap was 2 cm, and the width was 7 cm. Before the experiments, the subjects wore their customized sensing bands on the outside of their clothes and adjusted the bandage based on their own feelings. In the experiment, the subjects sat in the chair according to their own comfort (as shown in the left side of Figure 2). They were instructed to perform nine types of motion, including relax (R), wrist flexion/extension (WF/WE), wrist pronation/supination (WP/WS), wrist radius/ulna deviation (RD/UD), fist (F) and palm (P). When in the relaxed state, the subjects were asked to keep their hand in the neutral position and totally relaxed (with the palm being almost vertical to the ground). For each motion pattern except relax, five trials of the measurement were carried out. In each trial, the subject started from the relaxed state (R) and performed the corresponding motion to their maximum extent following the instructions. After maintaining the motion for a few seconds (about 10 s), the subject returned to the relaxed state and remained still for a further 5 s. The data from the first few seconds and the last few seconds of each trial were recorded as relax (R). The capacitance signals (six channels in total) and the IMU on the back of the palm were simultaneously sampled at 100 Hz and stored in the computer. After the experiment, nine motion patterns (R, WF, WE, WP, WS, RD, UD, P and F) with 16 transitions (R↔WF, R↔WE, R↔WP, R↔WS, R↔RD, R↔UD, R↔P, and R↔F) were investigated.

**Table 1:** Detailed information for seven healthy subjects (S1–S7).

	**Weight (kg)**	**Height (cm)**	**Measured arm**	**FL (cm)**	**FC (cm)**
S1	75	181	R	24	25
S2	64	177	R	23	23
S3	65	165	R	21	25
S4	90	180	R	24	25
S5	78	183	R	27	25
S6	85	185	R	27	26
S7	65	166	R	24	26

#### 2.2.2. Experiments with external disturbances

***Re-wearing the sensing band***: In addition to the motion recognition experiment, we tested the system performance with external disturbances. The aim of the experiment (Exp 2) was to test the recognition performance when re-wearing the sensing band. In this experiment, one subject was employed. The male subject was 23 years old, had a height of 181 cm, weighed 76 kg, and had a FL of 24 cm and FC of 25 cm. The sensing band was reshaped onto his right forearm. There were two experiment sessions. In each session, the subject performed the motion tasks according to the protocol of Exp 1. The two experiment sessions were measured on two different days. Before the experiment in each session, the subject was asked to wear his sensing band based on his own comfort.

***Different postures in relax***: In upper-limb motion recognition, postures of the arm also exerted an influence on the recognition performance (Khushaba et al., 2016). In this study, nine motion patterns were investigated, including six wrist joint motions, two gestures and relax. We carried out an experiment (Exp 3) on one male subject with varying wrist angles during the relaxed state (R). The subject was 29 years old, had a height of 181 cm, weighed 76 kg, and had a FL of 24 cm and a FC of 25 cm. The measured arm was the right forearm. The motion patterns were the same as that of Exp 1. During relax, the subject was asked to place his hand with a random tilt angle to the directions of the flexion/extension/pronation/supination/ulna deviation/radius deviation. The motion range of each trial was chosen based on his own thoughts and was below his maximum extent. The experiment setups were the same as Exp 1.

***Accumulated training test***: During on-line recognition, new data can also be added into the training data set. We therefore initially tested the performance with accumulated training. In this experiment (Exp4), one male subject was recruited. The subject was 23 years old, had a height of 185 cm, weighed 85 kg, had a FL of 27 cm and a FC of 26 cm. His right forearm was measured. The motion tasks were WF, WE, WP, WS, RD, UD, P, F, and R. In each trial, the experiment protocol was the same as that of Exp 1. After five trials of the measurement, the recorded signals were regulated and trained with the QDA classifier. With the classifier model, the new data from the sixth trial went through on-line recognition. The data for each new trial were also stored with all the previously measured data as the training data set and trained for a new classifier model. The accumulatively trained model was used to recognize the data of the next trial. In the experiment, we measured 15 trials for all of the motion patterns.

### 2.3. Recognition methods

#### 2.3.1. Data preprocessing

**Figure 3:**
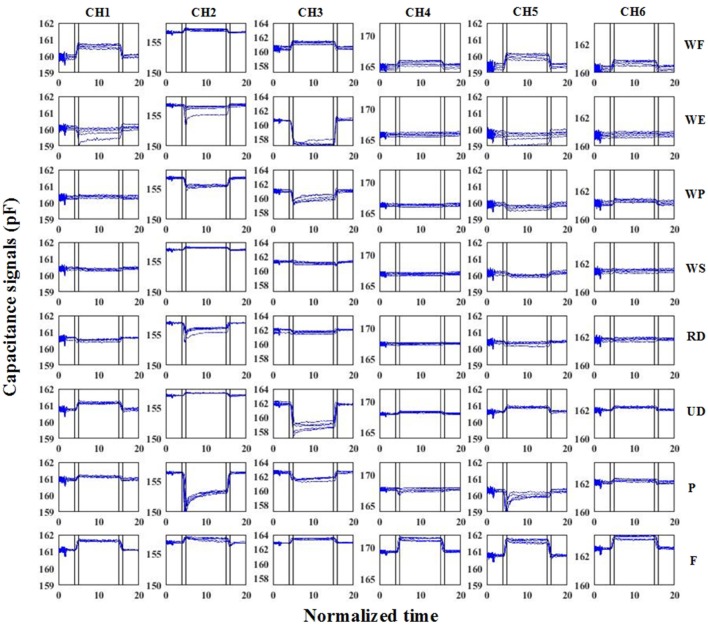
Raw capacitance signals of S6. In the subfigures, each column represents one signal channel, while each row represents one motion pattern. The blue lines are the filtered capacitance signals. The black vertical lines denote the motion transition periods. The vertical axis represents the absolute capacitance signals. The signals of all of the trials were plotted in the same sub-figure. The horizontal axis represents the normalized time.

The capacitance signals reflected the muscle shape changes during motion. As shown in Figure 3, the absolute capacitance values changed between several pFs. Using power spectrum analysis, there were high frequency noises in the raw capacitance signals. We used a 4-order Butterworth low-band-pass filter to remove the noise. The cut-off frequency was 5 Hz. The filtered signals clearly reflected the characteristics of the different motions. In this study, we designed a machine learning based method for discrete motion recognition. The filtered capacitance signals had to go through the data segmentation procedure. We used sliding windows to segment the data. In the method, a fixed-length window slid from the beginning to the end of the capacitance signals. The features were calculated on each window. The upper-limb motions investigated in this study involved the wrist joint motions of 3-DOF and two basic hand gestures. As shown in Figure 3, the signals were non-periodic, and the absolute values were correlated with the motion pattern itself. In the initial attempts, we selected four time-domain features that could characterize the capacitance signals. The features were AVE(**x**), STD(**x**), TAN(**x**) and MAX(**x**), in which **x** was the data of each sliding window of one channel. AVE(**x**) was the average value, STD(**x**) was the standard deviation, MAX(**x**) was the maximum value, and TAN(**x**) = [**x**(end)-**x**(1)]/WinLen, represented when WinLen was the length of the sliding window, **x**(end) and **x**(1) were the last and the first value of the sliding window, respectively. The feature calculation was repeated on all of the signal channels (six in total). The features of all the channels were serially connected as a feature vector for the subsequent analysis.

#### 2.3.2. Automatic labeling

The purpose of the automatic labeling was to automatically label the experiment data to corresponding motion patterns. In each experiment trial, the subject started with holding the relax (R) position for a few seconds and performed the instructed motion patterns. After continuing the motion pattern for about 10 s, the subject moved back to the relaxed state. Therefore, in each trial there were two motion transitions. We used a supervised learning method in which the classes (i.e. motion patterns) of the samples (feature vectors calculated from capacitance signals) had to be labeled as actual classes during the training procedure. Compared with manually labeling the experiment data, the automatic labeling method could increase the efficiency of the experiments.

**Figure 4:**
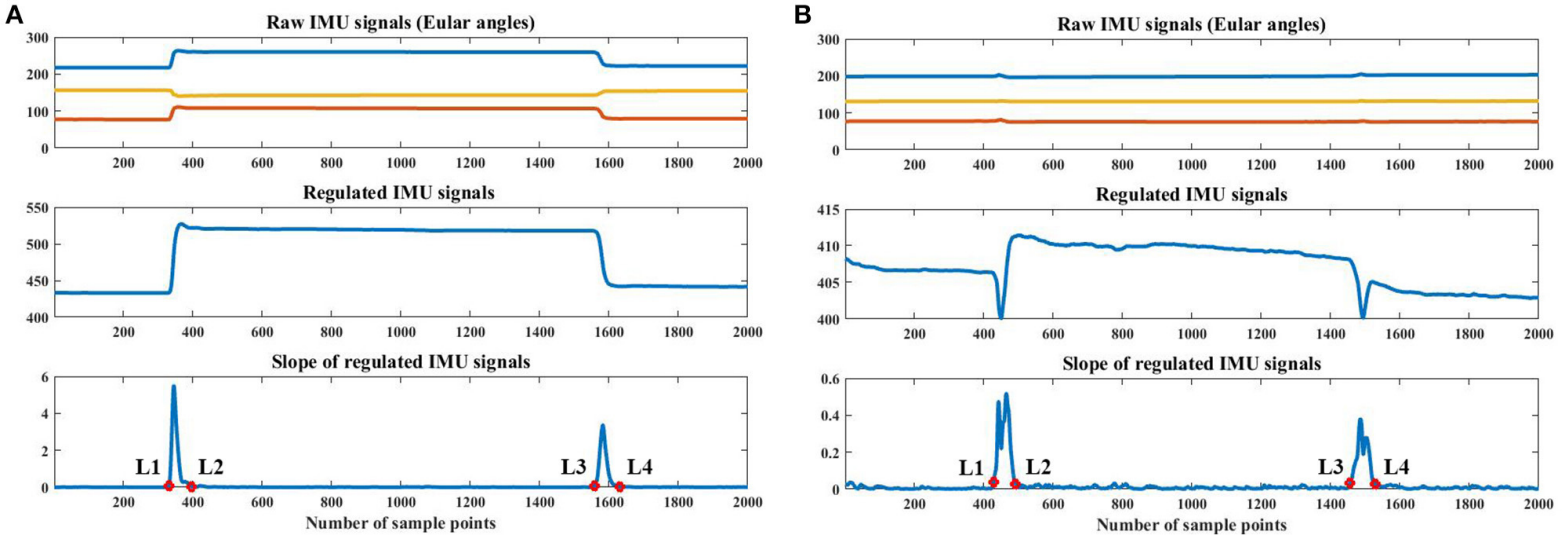
Automatic labeling based on IMU signals. **(A)** shows the IMU signals of the first trial of wrist pronation, and **(B)** shows those of the first trial of fist (F). In each subfigure, the top plot is the raw IMU signals with 3-axis Eular angles, the middle plot is the regulated IMU signals, and the bottom plot is the slope of the regulated IMU signals and the automatic labels (red circles). In the figures, L1, L2, L3 and L4 denote the automatically calculated labels. Data were collected from S1.

The signals of an IMU were used as an input into the automatic labeling algorithm. As shown in Figure 2, we placed an IMU circuit on the back of the palm of each subject. The IMU board integrated a gyroscope and an accelerometer. The 3-axis Eular angles (pitch, roll and yaw) were the output of the IMU circuit and were input into the following algorithm. The algorithm worked off-line after each experimental trial was finished. During the experiment for each subject, 3-axis Eular angles of the palm were recorded, as shown in the top plot of the Figure 4A. There were three main procedures in the algorithm, these were signal regulation (Procedure 1), extremum value calculation (Procedure 2) and threshold detection (Procedure 3). The goal of the method was to correctly identify the boundaries (L1, L2, L3, L4) between relax (R) and the corresponding motion pattern. As shown in the bottom plots of Figure 4, L1 and L2 indicated the time points that the motion transited from R to the corresponding motion patterns, with L1 being the initiation and L2 the termination. L3 and L4 indicated the motion switched from the corresponding motion pattern to R, with L3 being the initiation and L4 the termination.

***Procedure1***: In this procedure, we regulated the raw IMU signals to make them change in the same direction. If the absolute values decreased when the subjects transited from R to the target motion, the values of the channel would be flipped, based on the average value of the trial. We then summed up the 3-axis flipped signals and obtained the regulated IMU signal (as shown in the middle plot of Figure 4A).

***Procedure2***: In this procedure, we extracted the slope information of the regulated IMU signal and calculated the positions of the two-extremum values. There were two situations in this procedure. In the first situation, for most of the motion patterns investigated in this study, the slope of the regulated IMU signal during the two motion transitions was obviously larger than the other parts (as shown in the bottom plot of Figure 4A). The two maximum values were calculated from the slope of the regulated IMU signal (denoted by *S*). The extremum values were calculated through the 2nd-order differentials. If *d*(*i*) > 0 and *d*(*i*+1) < 0, then *i* would be one maximum value, where *d*(*i*) was the *i*−*th* point of differentials of *S*. In the second situation, the changes in the Eular angles were not as obvious for motion patterns such as palm (P) and fist (F). The maximum values of the same motion transition period may not be as unique (as shown in the bottom plot of Figure 4B). We designed an iterative method to calculate the positions of the extremum values. Firstly, all of the candidates of the maximal extremum values were calculated using the 2nd-order differentials (mentioned above) and were sorted by descending order. The sorted positions were expressed as *M*_*j*_, with *j* denoting the *j*−*th* maximum values. Secondly, we defined a duration threshold (*Duration*) to determine whether the extremum values were noises. The logic was expressed by Algorithm1, where *P*_1_ and *P*_2_ were positions of the two maximum values for the two transition periods.

***Procedure3***: In this procedure, L1, L2, L3, and L4 were identified by threshold detection. The threshold was extracted from the slope of the regulated IMU signal (S), and it was set to be the standard deviation of the middle 2 s of the trial, in which the motion was static. The values that went across the threshold were regarded as the candidates. To finally determine the boundaries, the points that were closest to the positions of the maximum values (*P*_1_ and *P*_2_ calculated from ***Procedure2***) were selected as the boundaries (as shown in the bottom plots of Figure 4).

**Algorithm 1:**
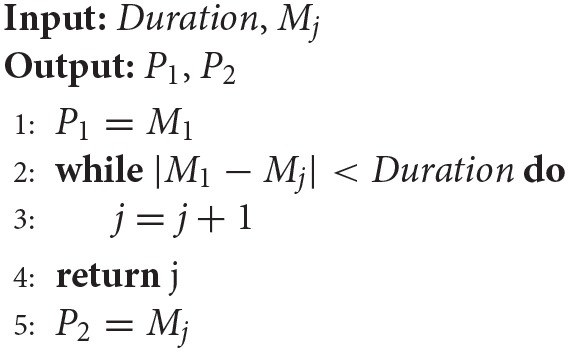


#### 2.3.3. Recognition

In this study, the supervised learning method was used for motion recognition. Firstly, the feature vectors were input into the classifier for training. After the model was fitted, the tested features were used to evaluate the performance. We used the quadratic discriminant analysis (QDA) method as the classifier. In QDA, the data distribution in the feature space was assumed to be a multivariate Gaussian distribution for each motion pattern (class). A mean vector and a covariance matrix were trained for each pattern based on the training data. In our previous studies (Zheng et al., 2014; Zheng and Wang, 2017), QDA was proved to be effective for the processing of capacitance signals with a proper computational load.

### 2.4. Evaluation methods

In this study, *N*-fold cross-validation (LOOCV) was used to evaluate the recognition method. In LOOCV, the data of one fold served as the testing set, while the rest data were used for training. The procedure was repeated for *N* times until all the data were used for the testing set. In this study, we measured five trials of data for each upper-limb motion pattern. *N* was therefore set to be five. The recognition decision could be made on each sliding window. The recognition accuracy (RA) was defined as:

(1) $$\begin{array}{r} RA=\frac{N_{correct}}{N_{total}}\times 100\% \end{array}$$

in which *N*_*correct*_ was the number of correct motion recognition decisions and *N*_*total*_ was the total number of testing data. As mentioned above, with automatic labeling method, there were four boundaries in each trial, i.e., L1, L2, L3 and L4. The data between L1 and L2 and between L3 and L4 were motion transition periods. Therefore, the decisions of the periods were not calculated in *RA*. To evaluate different upper-limb motion pattern, we used confusion matrix to illustrate the recognition performance of certain motion patterns. The confusion matrix was defined as:

(2) $$\begin{array}{r} C=\left(\begin{array}{cccc} c_{11} & c_{12} & \ldots & c_{1M}\\ c_{21} & c_{22} & \ldots & c_{2M}\\ \ldots & \ldots & \ldots & \ldots \\ c_{M1} & c_{M2} & \ldots & c_{MM} \end{array}\right) \end{array}$$

where each element is defined as:

(3) $$\begin{array}{r} c_{ij}=\frac{n_{ij}}{{\bar{n}}_{i•}}\times 100\%. \end{array}$$

*n*_*ij*_ is the number of testing data in class (motion pattern) *i* recognized as class *j* and ${\bar{n}}_{i•}$ is the total number of testing data in class *i*. A higher value of *c*_*ij*_ (*i*≠*j*) denotes that it is easier for class *i* to be misclassified as class *j*. Therefore, higher values on the diagonal indicates better recognition results. *M* is the number of classes to be calculated. There was difference of testing data sizes among the motion patterns. To investigate the average recognition accuracy across all the motion patterns *RA*_*c*_, we calculated the average value of the trace of the confusion matrix, which was expressed as:

(4) $$\begin{array}{r} RA_{c}=\frac{1}{M}\overset{M}{\underset{i\;=\;1}{\sum }}c_{ii}. \end{array}$$

*c*_*ii*_ was the value of the diagonal in the confusion matrix.

In addition to the recognition accuracy, we investigated the delay of the recognition system during motion transitions. In each trial of Exp 1, L1 and L3 were regarded as the time points that initiated the motion transitions (*T*_*i*_), while L2 and L4 were the termination of the transitions (*T*_*t*_). We calculated the difference between the recognized time points (*T*_*r*_) of transitions and the actual time points (labeled ones). The time points were referred to as *T*_*r*_, followed by ≥10 successive correct recognition decisions for the upcoming motion pattern. We named the differences as the prediction time (*PT*1 and *PT*2), which were expressed as *PT*1 = *T*_*r*_−*T*_*i*_ and *PT*2 = *T*_*r*_−*T*_*t*_. Positive values indicated the recognized transitions lagged behind the actual ones, while negative values indicated advanced decisions.

### 2.5. Confounding factors

#### 2.5.1. Sliding window length

Large sliding windows can extract more motion information but lead to longer latency. The influence of the sliding window length on recognition performance was evaluated. The window lengths from 50 ms to 300 ms with a 50ms interval were investigated. For each window length, the average recognition accuracies across the subjects were calculated with a 5-fold LOOCV. To evaluate the statistical significance, one-way repeated measure analysis of variance (ANOVA) was used, and the significance value was 0.05 (α = 0.05). In the one-way repeated measure ANOVA, the independent factor was a sliding window length and the dependent factor was the average recognition accuracy.

#### 2.5.2. Training data size

We evaluated the influence of the training data size on the recognition accuracy. In each subject experiment there were five trials. We used cross validation (CV) to evaluate the performance. We calculated the recognition accuracy with 5-fold LOOCV, 3:2 CV and 2:3 CV. The 5-fold LOOCV was the same as that mentioned above. In 3:2 CV, data from three of the trials was used for training and the remaining two trials for testing. In 2:3 CV, data from two of the trials were used for training and three trials for testing. The procedure was repeated until all the combinations were calculated. The average results across the combinations were regarded as the recognition accuracy. The sliding window length was used with the window selected in the procedure of the sliding window length.

#### 2.5.3. Motion pattern

We evaluated the influence of the motion patterns on recognition accuracy. Four combinations of motion patterns were taken into consideration. Combination 1: all of the nine motions were calculated (8 Motions + R for short). Combination 2: eight motions were calculated with the data of R removed (8 Motions for short). Combination 3: seven motion patterns were calculated with P and F removed (Wrist motions + R). Combination 4: seven motion patterns were calculated with RD and UD removed (6 Motions + R). Combination 5: five motion patterns were calculated including WF, WE, WP, WS, and R. The recognition accuracies of Combination 1 were compared with the other three combinations. A pair *t*-test was conducted for analysis of the recognition accuracies. The significance value was 0.05 (α = 0.05).

#### 2.5.4. External disturbances

For Exp 2, we used two cross validation (CV) methods to evaluate the recognition accuracy. In the 1:1 CV, the data of one experimental session was used for training and the other for testing. The procedure was repeated two times and averaged. The second method was 6:4 CV. In this method, data of one session plus one trial of the other session were used for training. The rest of the data from the session was used for testing. The procedure was repeated 10 times until all the combinations were tested. For Exp 3, recognition accuracy of the 5-fold LOOCV was investigated. For Exp 4, after 5 trials of measurement, the data of the new trial was on-line recognized. The QDA model was accumulatively trained with all of the previously measured data. As we measured 15 trials for nine motion patterns, there were 10 groups of recognition accuracies in total.

## 3. Results

### 3.1. Sliding window length and training data size

The average recognition accuracies (mean ± std) across the subjects with different sliding window lengths (from 50 to 300 ms) were 92.30 ± 4.54%, 92.32 ± 4.53%, 92.27 ± 4.57%, 92.14 ± 4.63%, 92.05 ± 4.67%, and 92.03 ± 4.66%. The highest recognition accuracy occurred at the 100 ms window length. The accuracies began to decrease with the increase of window length after 100 ms. We conducted one-way repeated measure ANOVA to test the influence of the sliding window length on the recognition accuracy. Mauchly's Test of Sphericity indicated that the assumption of sphericity had been violated, $\chi_{\left(14\right)}^{2}$ = 49.303, *p* < 0.0005, and therefore, a Greenhouse-Geisser correction was used. There was a significant effect of sliding window length on average recognition accuracies, *F*_(1.556, 9.337)_ = 7.172, *p* = 0.017. We therefore chose 100-ms window length for the subsequent analysis.

The results of the training data size were calculated with different CV folds and a sliding window length of 100 ms. The average recognition accuracies decreased to 81.01 ± 9.72% and 60.89 ± 12.76% for 3:2 and 2:3 CV, respectively. We therefore used the 5-fold LOOCV for the subsequent evaluation.

### 3.2. Overall recognition accuracy

**Figure 5:**
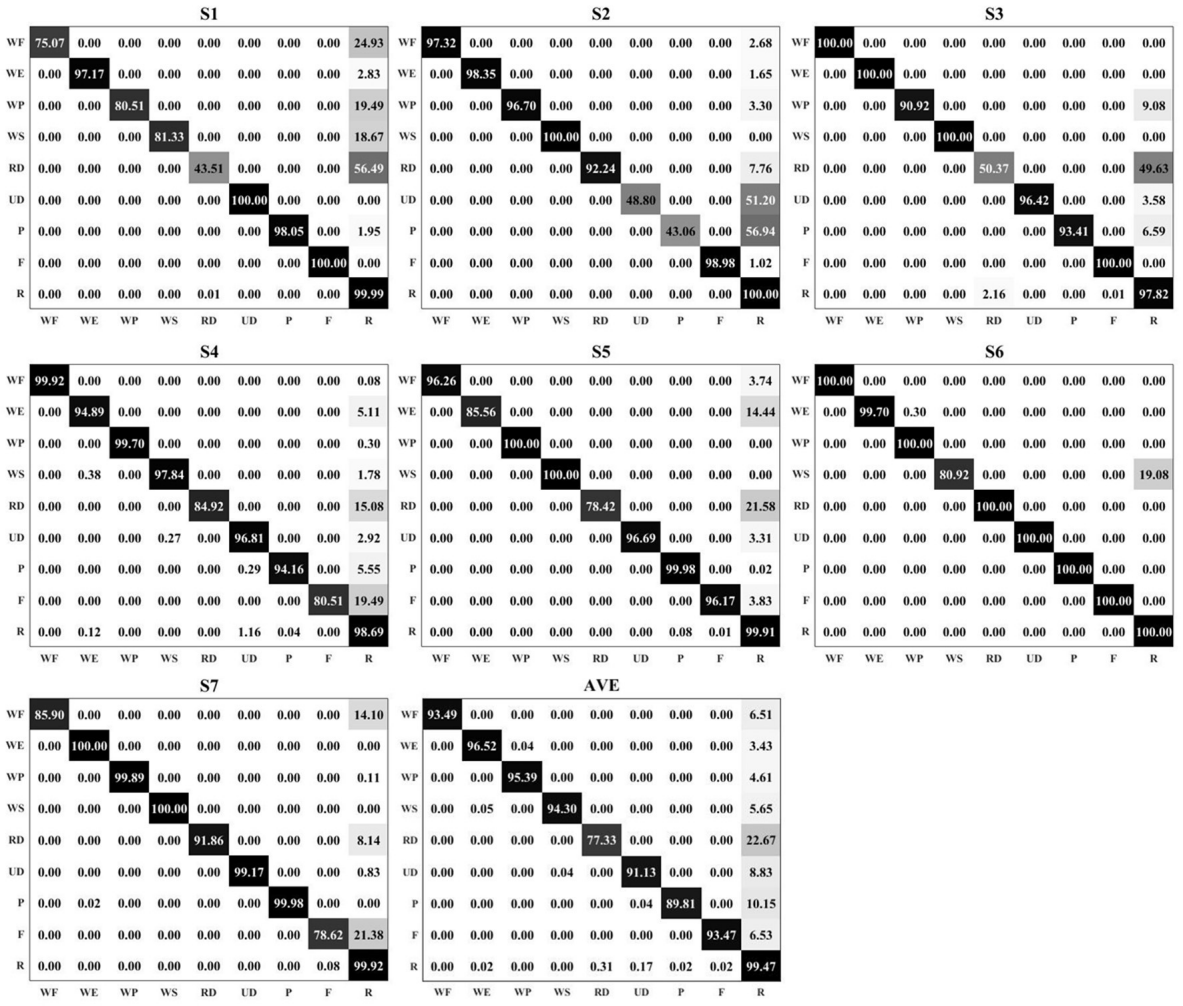
The confusion matrices for seven subjects. Each matrix represents the results of one subject, from *S*1 to *S*7, respectively. The matrix with the title AVE is the averaged results across the subjects. In the matrix, the darker color represents a higher value. All of the recognition accuracies and the mis-classification error rates are marked in the corresponding positions of the matrices.

The overall recognition accuracy is shown in Figure 5. Nine motions were measured on seven subjects. The sliding window length was 100 ms, and four time-domain features were selected for the feature set (as mentioned in section 2.3). The classifier was QDA, and 5-fold LOOCV was used to evaluate the performance. The average recognition accuracies (averaged across the diagonal of the confusion matrix) for each subject were 86.18, 86.16, 92.10, 94.16, 94.78, 97.85, and 95.04% from *S*1 to *S*7, respectively. By comparing the recognition accuracy in each confusion matrix, most of the errors were mis-classified as R. In the average results (using the confusion matrix with the title AVE in Figure 5), RD produced the lowest recognition accuracy (77.33%). During the experiments, the motion range of RD was much smaller than the other motions, and the signal changes were also small with respect to the relaxed state. Apart from RD, the system also produced lower recognition accuracies in UD, P, and F than the other motion patterns.

### 3.3. Prediction time of motion transitions

The prediction time of the system on motion transition recognition is shown in Tables 2, 3. In the tables, AVE_t was the average prediction time across the motion transitions, and AVE_s was the average prediction time across the subjects. The average PT1 across the subjects and the motion transitions was 480.29 ms and the average PT2 was -253.05 ms. The system produced around a 480-ms delay to the initiation of the transition, while it could successfully recognize the upcoming motion pattern at around 253 ms before the transition finished. The motion transitions took place between R and the corresponding motion patterns. The transitions can be categorized into two types, these are: Type 1: R → corresponding motion pattern; and Type 2: corresponding motion pattern → R. For the two transition types, the average PT1s were 346.67 ms and 613.89 ms for Type 1 and Type 2, respectively. The average PT2s were -333.32 ms and -172.79 ms for Type1 and Type2, respectively. We conducted a pair *t*-test (α = 0.05) to compare the difference in the prediction times between the two types of transitions. The values of PT1 and PT2 for all the subjects were used. *t*_(111)_ = −5.892, *p* < 0.0005. The results showed that the transition type had a significant effect on the prediction time.

**Table 2:** PT1 of the motion transitions (ms).

	**R → WF**	**R → WE**	**R → WP**	**R → WS**	**R → RD**	**R → UD**	**R → P**	**R → F**	**WF → R**	**WE → R**	**WP → R**	**WS → R**	**RD → R**	**UD → R**	**P → R**	**F → R**	**AVE_t**
S1	372	318	390	356	296	268	212	284	408	642	468	580	330	728	738	850	452.50
S2	618	336	260	290	372	350	368	210	248	602	490	764	300	208	208	954	411.13
S3	268	184	288	288	1492	258	154	228	794	840	686	724	118	578	774	810	530.25
S4	202	222	238	298	794	228	594	212	774	496	420	648	522	578	534	648	463.00
S5	232	422	292	506	292	254	228	192	824	562	684	756	262	596	934	886	495.13
S6	716	330	248	552	228	252	134	298	706	724	826	720	700	854	1120	912	582.50
S7	438	332	492	364	598	380	228	158	546	344	360	656	262	400	658	624	427.50
AVE_s	406.57	306.29	315.43	379.14	581.71	284.29	274.00	226.00	614.29	601.43	562.00	692.57	356.29	563.14	709.43	812.00	480.29

**Table 3:** PT2 of the motion transitions (ms).

	**R → WF**	**R → WE**	**R → WP**	**R → WS**	**R → RD**	**R → UD**	**R → P**	**R → F**	**WF → R**	**WE → R**	**WP → R**	**WS → R**	**RD → R**	**UD → R**	**P → R**	**F → R**	**AVE_t**
S1	-404	-386	-256	-284	-498	-708	-574	-456	-580	-350	-286	-376	-428	-298	-294	26	-384.50
S2	-182	-298	-324	-288	-222	-376	-518	-510	-466	-266	-194	-162	-404	-570	-708	-104	-349.50
S3	-372	-384	-254	-220	832	-358	-396	-462	-64	98	76	20	-962	-278	78	100	-159.13
S4	-302	-196	-126	-322	138	-480	-18	-588	-46	44	-38	-4	-460	-230	-104	44	-168.00
S5	-462	-358	-268	-246	-326	-436	-408	-776	50	-194	10	14	-602	-74	220	-36	-243.25
S6	-184	-360	-308	-80	-444	-484	-594	-480	-86	-82	150	10	-192	184	102	-50	-181.13
S7	-240	-454	-264	-332	-172	-312	-440	-446	-260	-542	-384	18	-480	-296	64	-34	-285.88
AVE_s	-306.57	-348.00	-257.14	-253.14	-98.86	-450.57	-421.14	-531.14	-207.43	-184.57	-95.14	-68.57	-504.00	-223.14	-91.71	-7.71	-253.05

### 3.4. Motion patterns

**Figure 6:**
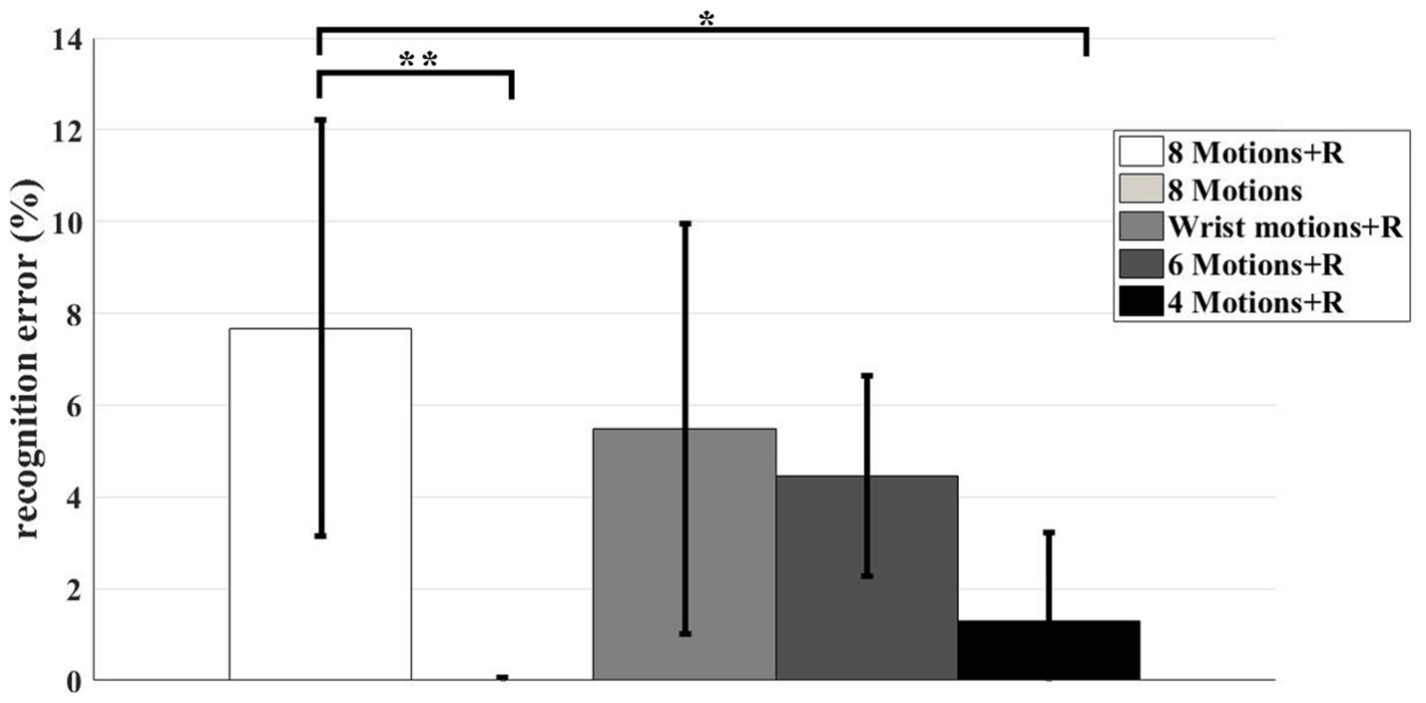
The recognition errors with different combinations of motion patterns. The bar charts were mean±std across seven subjects. The result of each combination was shown in different color, as shown in the legend. The asterisks indicate statistical significance. The signal asterisk indicates that *p* < 0.05, while the double asterisks indicate *p* < 0.01.

In this study, nine motions (including relax) were investigated. We found that the recognition accuracies were influenced by the motion types (as shown in Figure 6). The average recognition accuracy across the subjects of all the motion patterns used was 92.32 ± 4.53%. The highest recognition accuracy occurred when the data for R was removed. The accuracy was 99.98 ± 0.04%. For the rest of the three combinations, the accuracies were 94.51 ± 4.48%, 95.54 ± 2.19%, and 98.7 ± 1.94% for Combination 3, Combination 4 and Combination 5, respectively. We conducted a pair *t*-test to evaluate the influence of the motion patterns. The recognition accuracy of “8 Motions + R” was paired with that of the other four combinations. For the pair of “8 Motions + R” and “8 Motions,” *t*_(6)_ = −4.456, *p* = 0.004. For the pair of “8 Motions + R” and “Wrist motions + R,” *t*_(6)_ = −1.822, *p* = 0.118. For the pair of “8 Motions + R” and “6 Motions + R,” *t*_(6)_ = −2.29, *p* = 0.062. For the pair of “8 Motions + R” and “4 Motions + R,” *t*_(6)_ = −3.665, *p* = 0.011. Statistical significance of the motion patterns on recognition accuracies was found when the data of R were removed (Combination 2) and only the data of WF, WE, WP, WS, and R were recognized (Combination 5).

### 3.5. Results with external disturbances

The external disturbances investigated in this study seriously affected the recognition performances. For the results of Exp 2 (re-wearing of the sensing band inter-day), the recognition accuracies were 30.9% (1:1 CV) and 67.0% (6:4 CV). For the results of Exp 3 (different postures in relax), although trained with different postures in R, the recognition accuracy was still low (59.7% across nine motions). With R excluded, the average recognition accuracy increased to 94.8%. For Exp 4 (accumulated training test), we measured 15 trials of data for each motion pattern. The QDA model was trained after five trials of the experiment and the accumulated training and testing procedure was carried out from the sixth trial. The average recognition accuracies from the sixth trial to the 15th trial were: 23.01, 44.37, 63.98, 87.58, 98.89, 67.60, 96.50, 84.61, 93.28, and 99.64%. The recognition accuracies rapidly increased from the beginning to 98.89% (tenth trial). After the tenth trial, the low recognition accuracy also occurred in the 11th trial (67.60%). When we compared the detailed results, most of the errors were mis-recognized as R. For Exp 4, we investigated the computational cost of the recognition system. As mentioned above, we calculated four features for each signal channel. With the QDA classifier, nine 24 × 24 matrices and nine 24 × 1 vectors were trained with the sample data. There were three procedures for training, these were signal preprocessing, feature calculation and QDA model training. The training time was determined by the size of the training data set. We ran the training procedure ten times to show the worst case scenario (15 trials of data for training) for this study. The algorithms were implemented with MATLAB2016b on a computer with a CPU of Inter(R) Core(TM) i7-6500U (2.5 GHz frequency) and a RAM of 7.89 G. The average times were 17.54, 61.58, and 3.44 s for signal preprocessing, feature calculation and QDA model training, respectively.

## 4. Discussion and conclusion

In this study, we proved the feasibility of noncontact capacitive sensing for human upper-limb motion recognition. One obvious merit of the capacitive sensing method over sEMG sensing methods was that it produced accurate motion recognition results with the metal electrodes not being in contact with human skin. During the experiment, the sensing front-ends were worn on the outside of the subjects clothes, which relieved the difficulty of configuration before the measurements were taken. In the capacitive sensing system of this study, an equivalent plate capacitor was made up of the metal electrode (fixed on the inner surface of the sensing band), the human body and the cloth between them. The muscle deformations caused a change in the thickness of the cloth. According to the basic equations for the plate capacitor, if there are pressures between the human body and the sensing band, the capacitance signals will be positively correlated to the pressure of that spot within a proper range. However, as the capacitance signal changes were determined by the distance between the human body and the metal electrodes, not just the pressure, measuring the capacitance signals can obtain more sensitive information than only pressure signals (Honda et al., 2007).

Compared with the existing sEMG-based studies on upper-limb motion recognition, the recognition performances produced by noncontact capacitive sensing were at the same level. The first important performance was the recognition accuracy. Due to the difference in the motivation and processing methods, it is difficult to directly compare the recognition accuracy. One confounding factor accepted in many studies (Atzori et al., 2015, 2016) is the number of classes (motion patterns) to be analyzed. In our study, the average recognition accuracy was 92.32% with nine motion patterns. The accuracy increased to 95.54% with seven motion patterns and 98.70% with five motion patterns. According to a recent study (Atzori et al., 2016), the sEMG-based studies reached about 90–95% accuracy with 4–12 classes. The study suggested that the recognition accuracy of the noncontact capacitive sensing method was at the same level as that of sEMG-based studies. In addition to the recognition accuracy, we also investigated the prediction time of the transition periods. The average time delay to the initiation of the transitions, from R to the corresponding motion patterns was 346.67 ms. In the sEMG-based study performed by (Smith et al., 2011), the controller delay was referred to as the sliding window length and the authors reported that the optimal window length was between 150–250 ms. A more recent study (Wurth and Hargrove, 2014) reported a reaction time of around 600 ms on four forearm motion tasks. The time was defined as the duration between the target appearance to the user and the first successful recognition decision. Another important factor was the computational load during recognition which also affected the time response of the system. In this study, the dimension of the feature vector was 1 × 24. In on-line recognition, multiplications of around 1.25 × 10^6^ were needed before one decision was made. The computational load was acceptable for the current micro control units (MCU). With the MCU (STM32F767VGT6, STMicroelectronics, Co., Ltd.,) we used in the measurement system, the calculation could be finished within 6 ms. It enabled the system to update the recognition decisions in each sample interval (10 ms).

On the other hand, the limitations of the study should be noted. First, the capacitance signals were sensitive to the postures of the wrist joint during the relax position, which decreased the recognition accuracies. Although trained with varying postures (Exp 3), the recognition accuracy was still low (59.7%). The recognition accuracy increased to 94.8% when the R data was removed. The results suggested that the reduction of the recognition accuracy was caused by the feature distribution of the capacitance signals. The significant influence of the orientation of the forearm on recognition accuracies was also reported in sEMG-based studies (Khushaba et al., 2016). Secondly, current setups of the noncontact capacitive sensing approach cannot produce a stable recognition performance against the disturbances of re-wearing the sensing front-ends on different days. The cross validations between the two experiment sessions (with the re-wearing procedure between them) showed low recognition accuracies. Thirdly, the on-line recognition with accumulated training was initially tested in this study. It showed a trend that when more data was used in the training set, a higher recognition accuracy would be obtained. However, factors including the feature distribution changes over time, individual differences on multiple subjects, and so forth still need to be addressed. Future systematic studies are required for on-line recognition with the capacitive sensing method.

Despite the limitations, the capacitive sensing method is still a promising tool that is worth exploring. Future studies will be carried out in the following areas. Firstly, continuous estimation of upper-limb motion information with noncontact capacitive sensing will be explored. The recognition results in this study has proven the feasibility of the noncontact capacitive sensing approach on human upper-limb motion sensing. The results from varying the position during the relaxed state (Exp 3) also suggested that the capacitance signals are correlated to wrist joint positions. In continuous motion estimation, the physical significance (such as the correlations of the capacitance signals to the pressures and to the muscle morphological parameters) of the capacitance signals will be studied. Secondly, more extensive experiments will be carried out on real-time recognition. Methods that fuse capacitive sensing methods with other sensors (inertial sensors and force sensors) will be designed and evaluated. Robotic control based on capacitance signals will also be carried out. Thirdly, new materials will be used in the manufacture of the sensing front-ends. A soft elastic sensing band will be studied to cope with the individual differences in upper-limb shapes. We plan to address the problems of the re-wearing procedure on different days by using a softer material, to make the sensing front-end more stable on the human upper limb.

## Author contributions

QW and EZ designed research. EZ, JM, YL, and QW performed research. EZ analyzed data. EZ and QW wrote the paper.

### Conflict of interest statement

The authors declare that the research was conducted in the absence of any commercial or financial relationships that could be construed as a potential conflict of interest.

## Footnotes

^1^ Available online at: https://www.xsens.com/products/
